# Oncolytic Virus Infection Modulates Lysine Acetyltransferase in Gliomas: Comprehensive Analysis and Experimental Validation of KAT8 in Glioma

**DOI:** 10.1111/jcmm.70558

**Published:** 2025-04-21

**Authors:** Xiaofeng Yin, Zhen Hao, Qi Liu, Rui Ding, Laizhao Chen, Mingliang Jin, Songquan Wang

**Affiliations:** ^1^ Department of Neurosurgery Second Hospital of Shanxi Medical University Taiyuan China; ^2^ Department of Anesthesiology Taiyuan Central Hospital Taiyuan China

**Keywords:** apoptosis, glioma, KAT8, oncolytic virus, therapeutic target

## Abstract

Oncolytic virotherapy, which uses engineered viruses to selectively target tumour cells, has emerged as a potential treatment in glioma. However, how oncolytic virus infection modulates lactylation enzymes in gliomas remains unclear. The RNA‐seq data after oncolytic virus EV‐A71 infection on glioma cells was analysed to screen and lysine acetyltransferase 8 (KAT8) was selected. We analysed KAT8 expression in glioma tissues using data from The Cancer Genome Atlas (TCGA) and Genotype‐Tissue Expression (GTEx) databases. Associations between KAT8 expression and clinicopathological features were evaluated. Kaplan–Meier survival analysis and Cox regression models were used to assess the prognostic value of KAT8. Functional annotation analyses were performed to explore the KAT8‐related biological processes. Single‐cell RNA‐sequencing data were analysed to investigate cell‐type‐specific KAT8 expression. In vitro experiments were conducted to validate the effects of KAT8 knockdown on apoptosis in glioma cells. KAT8 was among the top 5 down‐regulated genes in glioma cells after oncolytic viruses EV‐A71 infection. KAT8 was significantly overexpressed in glioma tissues compared to normal brain tissues, with higher expression in lower‐grade gliomas. High KAT8 expression was associated with 1p19q co‐deletion, IDH mutation, and younger patient age. Notably, high KAT8 expression correlated with better overall survival in grade IV gliomas. Functional analyses revealed KAT8's involvement in neuronal development, mRNA processing, and apoptosis regulation. Single‐cell sequencing data revealed a negative correlation between KAT8 expression and apoptosis in glioblastoma cells. In vitro experiments confirmed the increased apoptosis in glioma cell lines following KAT8 knockdown. Our findings suggest that the regulatory pathway of oncolytic virus infection for lactylation enzymes may be a key pathway affecting gliomas. These findings highlight KAT8 as a potential prognostic biomarker and therapeutic target for gliomas.

## 1 Introduction

Gliomas are the most common primary malignant brain tumours, characterised by their heterogeneity and poor prognosis [1]. Traditional therapeutic approaches, such as surgery, radiation, and chemotherapy, offer only modest survival benefits, highlighting the need for novel strategies to improve patient outcomes. In recent years, oncolytic virotherapy has emerged as a promising alternative, leveraging engineered viruses that selectively infect and destroy tumour cells while sparing healthy tissues [2]. Among these, oncolytic viruses have shown particular potential in treating gliomas due to their ability to cross the blood–brain barrier and target tumour cells more specifically [3]. Our previous study also reported the mechanism by which oncolytic viruses regulate gliomas through ABCD3 [4].

Oncolytic virus (OV), a novel anti‐tumour therapeutic with wide‐ranging immunogenic properties, represents one of the most promising approaches for both preclinical and clinical cancer treatment [5]. A notable example is Teserpaturev, a genetically engineered HSV‐1 virus of the third generation, which enhances tumour specificity and induces a prolonged immune response [6]. An intratumoural mode of Teserpaturev injection reveals preliminary efficacy and manageable safety as a first‐line therapy for patients with gastric cancer, with a median overall survival rate of 28.8 months [7]. A critical aspect of glioma biology that impacts the efficacy of oncolytic virotherapy is the regulation of cellular metabolic pathways, including those involved in post‐translational modifications [8]. Among these, lactylation has emerged as a novel post‐translational modification that bridges the gap between cancer development and patient outcomes [9]. This study focused on KAT8, a key lactylation enzyme, and its potential role in glioma progression and prognosis.

Lactylation, a recently discovered post‐translational modification, has emerged as a crucial regulator of various cellular processes, including gene expression and metabolism [10]. This modification involves the addition of lactylation groups to proteins, particularly histones, which can significantly alter their function and interactions within the cells [11]. Recent studies have implicated lactylation in cancer development and progression, with growing evidence suggesting its involvement in glioma pathogenesis [12]. The potential role of lactylation in modulating gene expression and cellular metabolism in glioma cells has sparked considerable interest in the scientific community [13].

The enzyme KAT8, also known as MYST1 or MOF, has been primarily recognised for its histone acetyltransferase activity [14]. However, recent research has identified KAT8 as a potential lactylation regulator, warranting further exploration of its role in glioma biology [15]. The dual functionality of KAT8 in both acetylation and lactylation processes suggests a complex interplay between these post‐translational modifications in the context of glioma development and progression [16].

This study aimed to investigate the expression patterns of KAT8 under OV infection and in glioma tissues and also to evaluate its potential as a prognostic biomarker. Through comprehensive bioinformatics analyses of publicly available datasets, we examined KAT8 expression levels across different glioma grades and subtypes, assessed its correlation with patient survival, and explored its association with various clinicopathological features [17]. Additionally, we conducted function analyses to elucidate the potential molecular mechanisms underlying KAT8's role in glioma progression. Understanding these molecular mechanisms may provide new insights into glioma biology and potentially lead to novel therapeutic strategies targeting KAT8‐mediated pathways in glioma treatment [18].

## 2 Materials and Methods

### 2.1 Data Acquisition and Bioinformatics

Transcriptome analysis after infection of gliomas with OV Enterovirus A71 (EV‐A71) was conducted as we previously reported. Briefly, glioma CCF (astrocytoma) cells were infected with either the EV‐A71 BrCr strain or a mock strain, with a multiplicity of infection (MOI) of 1 for 60 min at room temperature. Total RNA extracted from CCF glioma cells was subjected to RNA‐seq using the Illumina HiSeq 2000 platform. After obtaining the raw data, FastQC was employed for quality assessment. Differentially expressed genes (DEGs) were identified using GFOLD version 1.1.4, with genes showing a GFOLD value ≥ 1 or ≤ −1 being considered significant.

### 2.2 Tissue Samples

A total of 5 formalin‐fixed paraffin‐embedded (FFPE) glioma tissue samples, including 1 pilocytic astrocytoma (WHO grade I), 2 anaplastic astrocytomas (WHO grade III), and 2 glioblastomas (WHO grade IV). The average age of the patients was 46.4 years (range, 35–82 years old). None of the patients had received any type of therapy prior to surgery. As a control, five non‐tumoral tissues (obtained from patients who underwent epilepsy surgery) were used to assess KAT8 expression. The Ethics Committee of Second Hospital of Shanxi Medical University and the Ethics Committee of Taiyuan Central Hospital previously approved the present study, and all the samples enrolled in the present study were unlinked and unidentified from their donors. All patients signed a consent form.

### 2.3 The Expression Analysis of KAT8

RNA‐sequencing data from The Cancer Genome Atlas (TCGA) were used, specifically from the TCGA‐GBM and TCGA‐LGG projects. A total of 701 samples were included in the analysis, including glioblastoma multiforme (GBM) and lower‐grade glioma (LGG) samples. The data were retrieved from the TCGA portal, processed using the STAR workflow, and the expression values were quantified in Transcripts Per Million (TPM) format. The clinical data for each sample were collected for subsequent analyses. To evaluate the differential expression of KAT8, samples were categorised into two groups based on gene expression levels. The low expression group (Low) consisted of the bottom 50% of samples (*n* = 350), while the high expression group (High) consisted of the top 50% of samples (*n* = 351). The DESeq2 package was used to perform differential expression analysis of raw counts of KAT8.

### 2.4 KAT8 Expression and Clinical Characteristics

Data on KAT8 gene expression and related clinical information for GBM and LGG patients were obtained from TCGA and Genotype‐Tissue Expression (GTEx) databases. Information regarding WHO classification, IDH mutation status, and 1p/19q co‐deletion was sourced from Ceccarelli et al.

### 2.5 Survival Prognosis Analysis

Overall survival (OS) and disease‐specific survival (DSS) were examined for survival analysis. GBM and LGG patients were categorised into high and low KAT8 groups based on the mean TPM value. R version 3.4.3, along with the survival packages, was utilised for survival analysis, Cox regression, and visual representations. Kaplan–Meier (K‐M) curves were employed to assess differences in survival time. Log‐rank tests were conducted, with *p*‐values below 0.05 considered statistically significant.

### 2.6 Functional Enrichment Investigation

Gene Set Enrichment Analysis (GSEA) was employed to explore potential biological functions associated with KAT8. Gene Ontology (GO) terms and Kyoto Encyclopedia of Genes and Genomes (KEGG) pathways were analysed for functional enrichment using a gene clustering analyser implemented in R. GO terms encompass three aspects: biological process (BP), molecular function (MF), and cellular composition (CC). KEGG pathway analysis provided insights into molecular interactions and signalling events related to KAT8. To ensure result reliability, an error detection rate (FDR) below 0.05 and a nominal *p* < 0.05 were required.

### 2.7 Single‐Cell Sequencing Data Evaluation

CancerSEA (http://biocc.hrbmu.edu.cn/CancerSEA/home.jsp), a database for single‐cell sequencing, was used to assess different functional states of cancer cells at the single‐cell level. Correlation data between KAT8 expression and various tumour functional states were retrieved from CancerSEA and visualised as a heatmap. T‐SNE diagrams of individual cells were directly obtained from the CancerSEA website.

### 2.8 Cell Lines, Plasmid and Virus Infection

Glioma cell lines including U87, A172 and immortalised human astrocytes (AS) cell line SVG p12 were purchased from Cell Bank of Chinese Scientific Academy (Shanghai, China). The above cell lines were cultured in Minimum Essential Medium (MEM) (Invitrogen, Carlsbad, CA) with 10% fetal bovine serum (Gibco, Life Technologies, CA). Cells were cultured at 37°C with 5% CO_2_ in a humidified incubator. The EV‐A71 BrCr strain was purchased from BioVector NTCC (ATCC VR‐784) and infected glioma cells when the culture cells were 70%–80% confluent. To rescue the expression of KAT8, a CDS sequence of KAT8 was cloned into a pLeGFP‐N3 vector. Then the glioma cells were transfected with the pLeGFP‐N3‐Kat8 plasmid. After 48 h post‐infection, the expression levels of KAT8 were assessed by Western blotting. The qPCR Primers for KAT8 were, 5′‐CGA GTA CTG CCT CAA ATA CAT GA‐3′ and 5′‐GCC ATC CAC TTC ATA CAC AGA‐3′. The antibodies used are as follows: Rabbit polyclonal Anti‐KAT8 / MYST1 / MOF antibody [EPR15803] was purchased from Abcam (USA). Rabbit polyclonal Anti‐actin was purchased from Beyotime (China).

### 2.9 Apoptosis Assay

Cells were trypsinised without EDTA and washed twice with phosphate‐buffered saline (PBS). Apoptosis was evaluated using the Annexin V‐APC/PI Apoptosis Detection Kit (#KGA1030‐50, KeyGen, Nanjing, China) according to manufacturer instructions, followed by flow cytometry analysis. Experiments were conducted in triplicate and repeated three times.

### 2.10 Western Blot

Proteins were isolated using RIPA buffer (Invitrogen) containing a protease inhibitor cocktail (Thermo Scientific Pierce, CA, USA). The samples were separated by SDS‐PAGE using 8% gels for 2 h. The separated proteins were then transferred onto PVDF membranes (Millipore, MA, USA). After blocking with 5% non‐fat milk for 1.5 h, the membranes were exposed to specific primary antibodies at 4°C overnight. Following PBST washing, the membranes were treated with corresponding secondary antibodies at room temperature for 2 h. The immunoreactive signals were visualised using the ECL detection system.

### 2.11 Statistical Analysis

Differences between the two groups were analysed using unpaired *t*‐tests, with data presented as mean ± standard deviation. Spearman's rank correlation coefficient was used to assess correlations between groups. The relationship between patient prognosis and KAT8 expression levels was evaluated using the Kaplan–Meier method. Statistical significance was set at *p* < 0.05.

## 3 Results

### 3.1 KAT8 Is Decreased During Oncolytic Virus Infection in Glioma

Our previous study reported a transcriptome analysis of glioma cells under oncolytic viruses EV‐A71 infection. Based on this, we re‐performed a transcriptome analysis to further investigate the lactylation effects of EV‐A71 infection on glioma cells. RNA‐seq data (PRJNA562271) were collected and following data acquisition, FastQC was used for quality control, confirming that the data met the criteria for further gene functional analysis. Gene expression levels were calculated using the TPM method and normalised with GFOLD. DEGs were identified by comparing EV‐A71‐infected groups to the mock infection group.

A total of 441 genes were up‐regulated (GFOLD ≥ 1), while 320 genes were down‐regulated (GFOLD ≤ −1) (Tables S1 and S2). Given the inherent ability of oncolytic viruses to infect and destroy tumour cells, the down‐regulation of certain genes in OV‐infected glioma could potentially contribute to the enhanced malignancy of gliomas. Notably, KAT8 was among the top five down‐regulated genes in glioma cells following EV‐A71 infection (Table S1). However, the expression and role of lactylation enzyme KAT8 in glioma have not been previously reported.

### 3.2 The Expression of KAT8 in Pan‐Cancer and Gliomas

**FIGURE 1:**
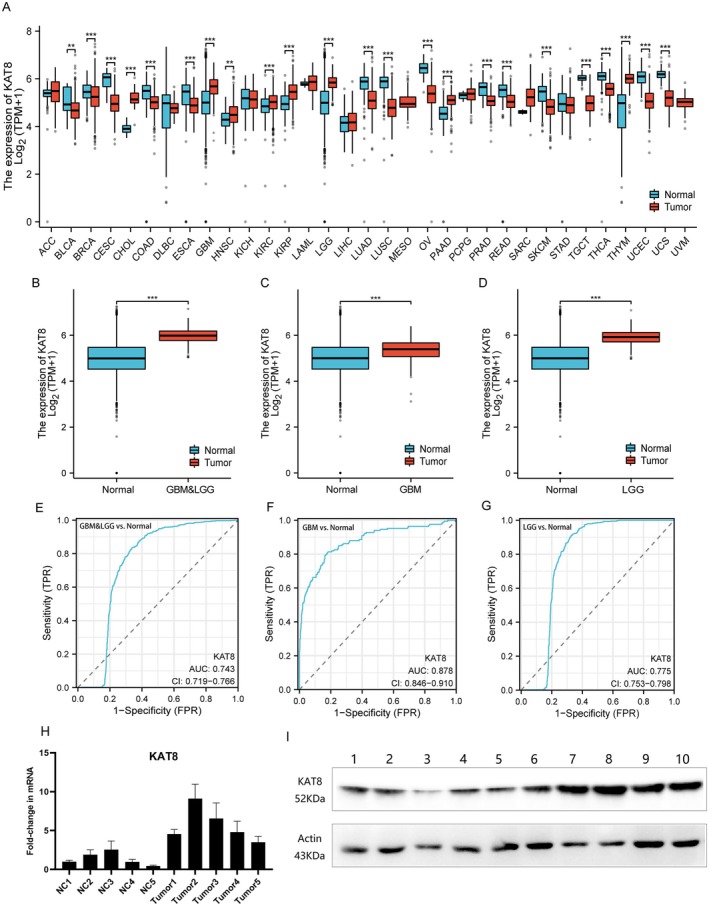
The expression level of KAT8 in tumours. (A) The expression of KAT8 at the pan‐cancer level. (B–D) The expression of KAT8 in glioma (including GBM and LGG) compared with normal tissues. (E–G) The ROC curves indicate KAT8 is a biomarker for diagnosis of glioma. The (H) mRNA and (I) protein expression of KAT8 in 5 glioma tissue specimens compared with 5 normal brain tissue samples.

Lactylation modification has been reported to be a key bridge between cancer development and potential patient outcomes. First, we analysed the expression of the lactylation enzyme KAT8 at the pan‐cancer level (Figure 1A). The results showed that the expression level of KAT8 was significantly higher in some tumour tissues than in normal tissues, such as in glioma (including GBM and LGG) (Figure 1B–D), CHOL, PAAD, and THYM. However, in other types of tumour tissues, its expression was lower than that in normal tissues, such as ESCA, LUSC, PRAD, and UCEC (Figure 1A). This suggests the heterogeneity of lactylation enzyme KAT8 in different tumour types as well as potential differential gene regulation mechanisms.

Analysis of the Receiver Operating Characteristic (ROC) curve indicates that the expression of the KAT8 gene demonstrates a commendable degree of accuracy in predicting the presence of disease among glioma patients, encompassing both GBM and LGG cases. The Area Under the Curve (AUC) values for the ROC curve consistently exceed 0.7 (Figure 1E–G), with an especially notable AUC value of 0.878 observed in GBM (Figure 1F). This value reflects the discriminative power of KAT8 expression in distinguishing glioma patients from healthy individuals. These findings underscore the potential of KAT8 as a reliable biomarker for glioma diagnosis, facilitating early detection and more effective disease management.

To further elucidate the expression and significance of KAT8 in glioma tissues, we randomly selected 5 clinical tissue specimens of varying WHO grades (1 WHO grade I sample, 2 WHO grade III samples and 2 WHO grade IV samples), as well as 5 normal brain tissue samples obtained from epilepsy surgery. Using RT‐qPCR and Western blotting, we assessed the expression of KAT8 in these clinical specimens. Notably, our results revealed that KAT8 was significantly up‐regulated in glioma tissues across all WHO grades, at both the mRNA and protein levels (Figure 1H,I). This finding is consistent with our previous bioinformatics analysis.

### 3.3 Associations Between KAT8 Expression and Clinicopathologic Variables in Glioma

**FIGURE 2:**
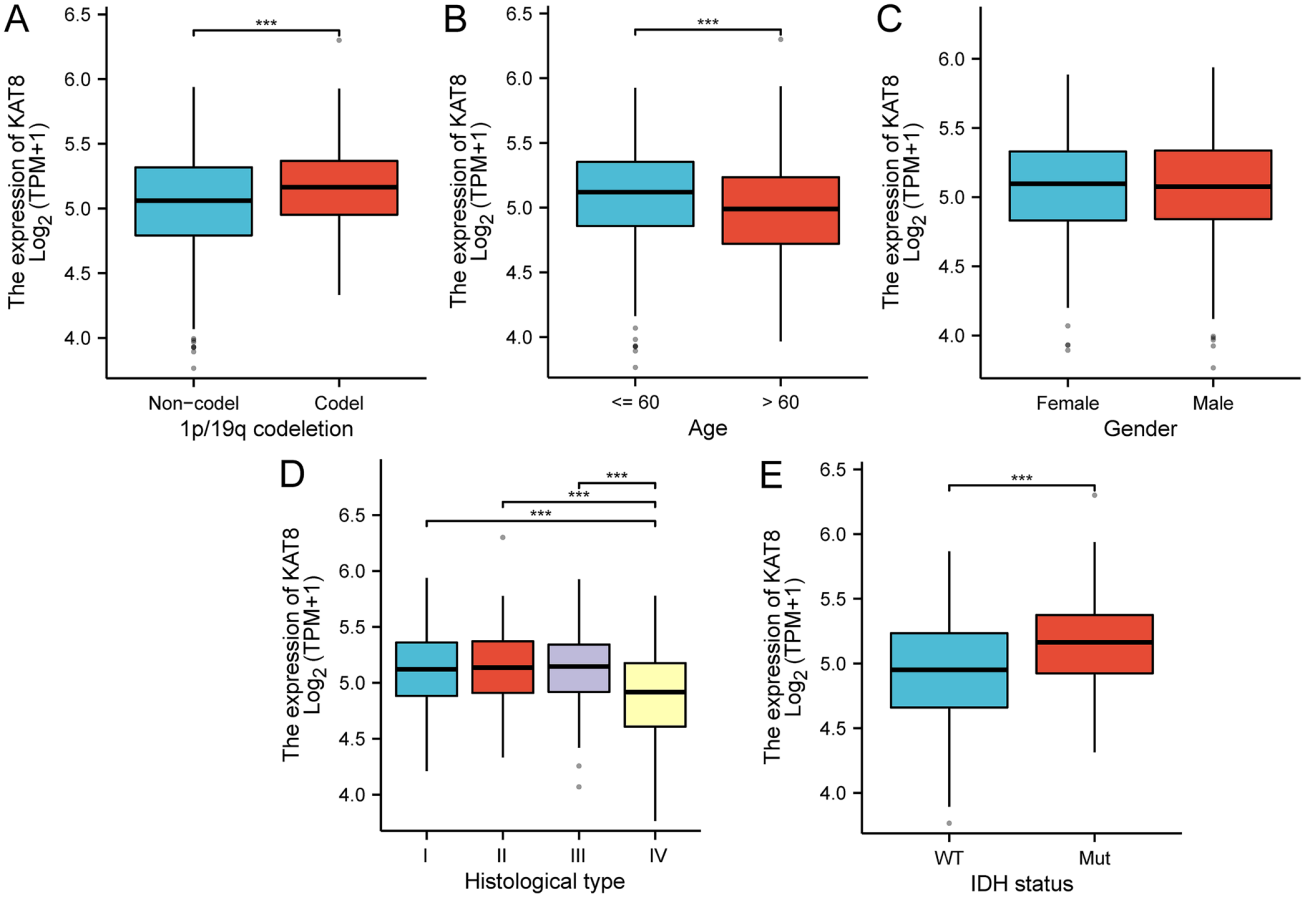
Associations between KAT8 expression and clinicopathological variables in glioma. The expression of KAT8 and its potential relationship with (A) 1p19q co‐deletion status, (B) age, (C) gender, (D) histological type, and (E) IDH status.

We investigated the expression of KAT8 and its potential relationship with various clinicopathological variables. We found that the expression of KAT8 was significantly higher in glioma patients with 1p19q co‐deletion (Codel group) than in those without 1p19q co‐deletion (Non‐Codel group) (Figure 2A). The Codel group demonstrated elevated KAT8 expression across various tumour grades. This observation is consistent with previous reports suggesting that 1p19q co‐deletion status is often associated with more favourable prognosis and better therapeutic responses in glioma. A significant difference in KAT8 expression was observed between glioma patients under age 60 and those over 60. Patients younger than age 60 exhibited higher expression levels of KAT8 than their older counterparts (Figure 2B). It could imply that KAT8 expression might be a factor in tumour progression or survival in younger glioma patients, further highlighting the need for age‐specific therapeutic approaches in glioma management. In contrast to the findings in age and genetic subgroup analyses, no significant difference in KAT8 expression was observed between male and female patients (Figure 2C). Our analysis showed that KAT8 expression was significantly lower in grade IV gliomas (glioblastoma) compared to grades I–III. Interestingly, no significant difference in KAT8 expression was observed among grades I, II, and III (Figure 2D). KAT8 expression was significantly higher in glioma patients with IDH mutations compared to those with wild‐type IDH (WT) (Figure 2E).

### 3.4 Prognostic Value of KAT8 in Glioma

**FIGURE 3:**
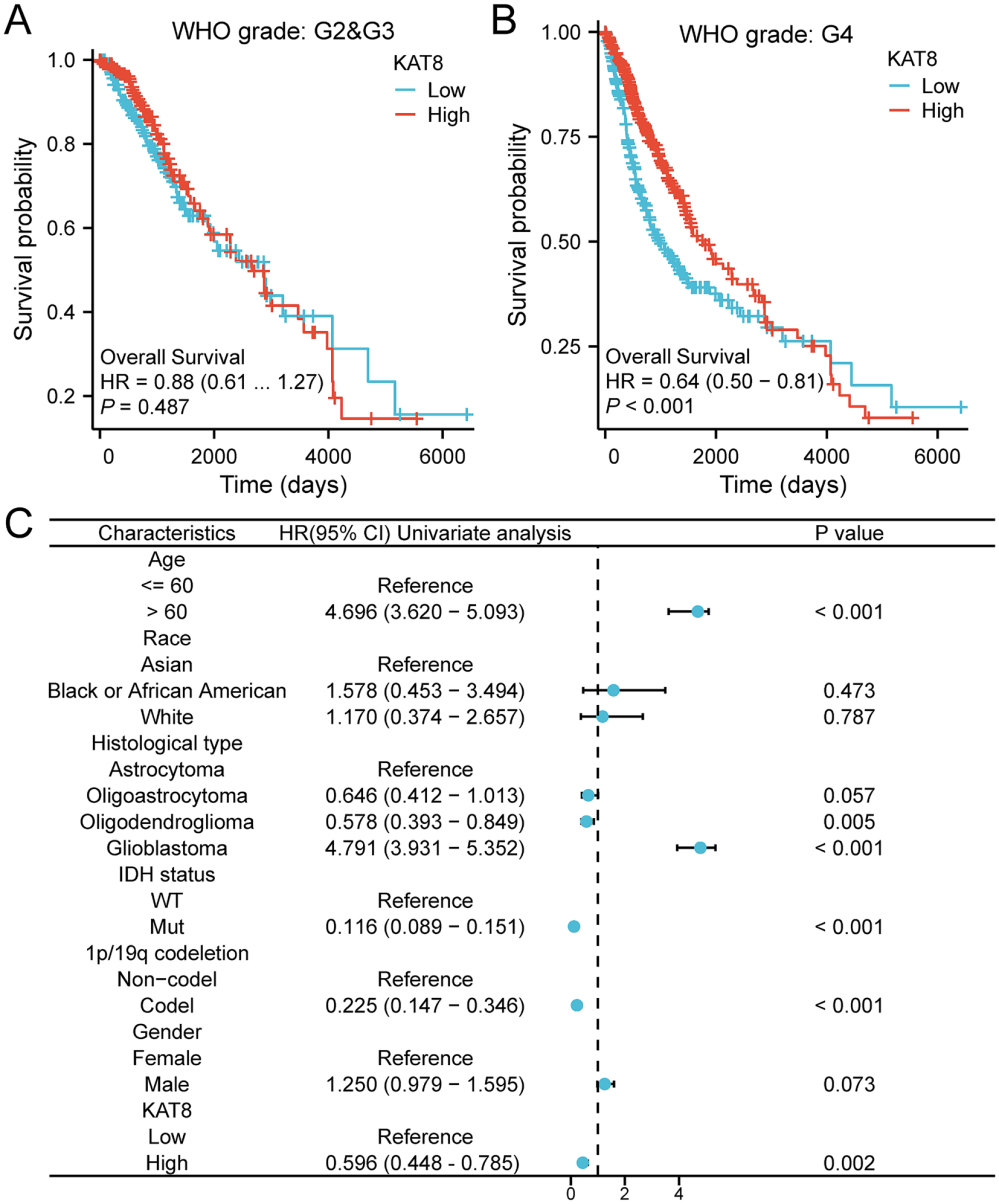
The association between KAT8 expression and prognosis in glioma. The association between KAT8 expression and prognosis in (A) grade II&III gliomas and (B) grade IV gliomas. (C) Forest map of OS with glioma patients based on multivariate Cox analysis.

We next investigated the relationship between KAT8 expression and prognosis in glioma patients. The patients were divided into two groups based on their KAT8 expression levels: high expression and low expression. KM survival analysis revealed no significant association between KAT8 expression and prognosis in grade II and grade III gliomas (Figure 3A). However, in grade IV gliomas, we observed a significant difference. High KAT8 expression was associated with a significantly better survival rate (Figure 3B), indicating that KAT8 high expression may serve as a protective factor in these patients.

To obtain a more comprehensive understanding of prognostic indicators, a multivariate Cox proportional hazards analysis was performed to investigate the effects of various clinical and molecular factors on the OS of glioma patients, with a focus on KAT8 expression. The analysis demonstrated that patients aged over 60 years showed a markedly higher risk of poor survival (HR = 4.696, 95% CI: 3.620–5.093, *p* < 0.001). The histological type of glioma also played a role in OS. Glioblastoma patients exhibited significantly worse survival outcomes when compared to those with other glioma subtypes, such as astrocytoma and oligodendroglioma. In contrast, the high expression of KAT8 was associated with a significantly better OS (HR = 0.696, 95% CI: 0.548–0.885, *p* = 0.002) (Figure 3C).

### 3.5 Functional Annotation Analysis of DEGs Related to KAT8

**FIGURE 4:**
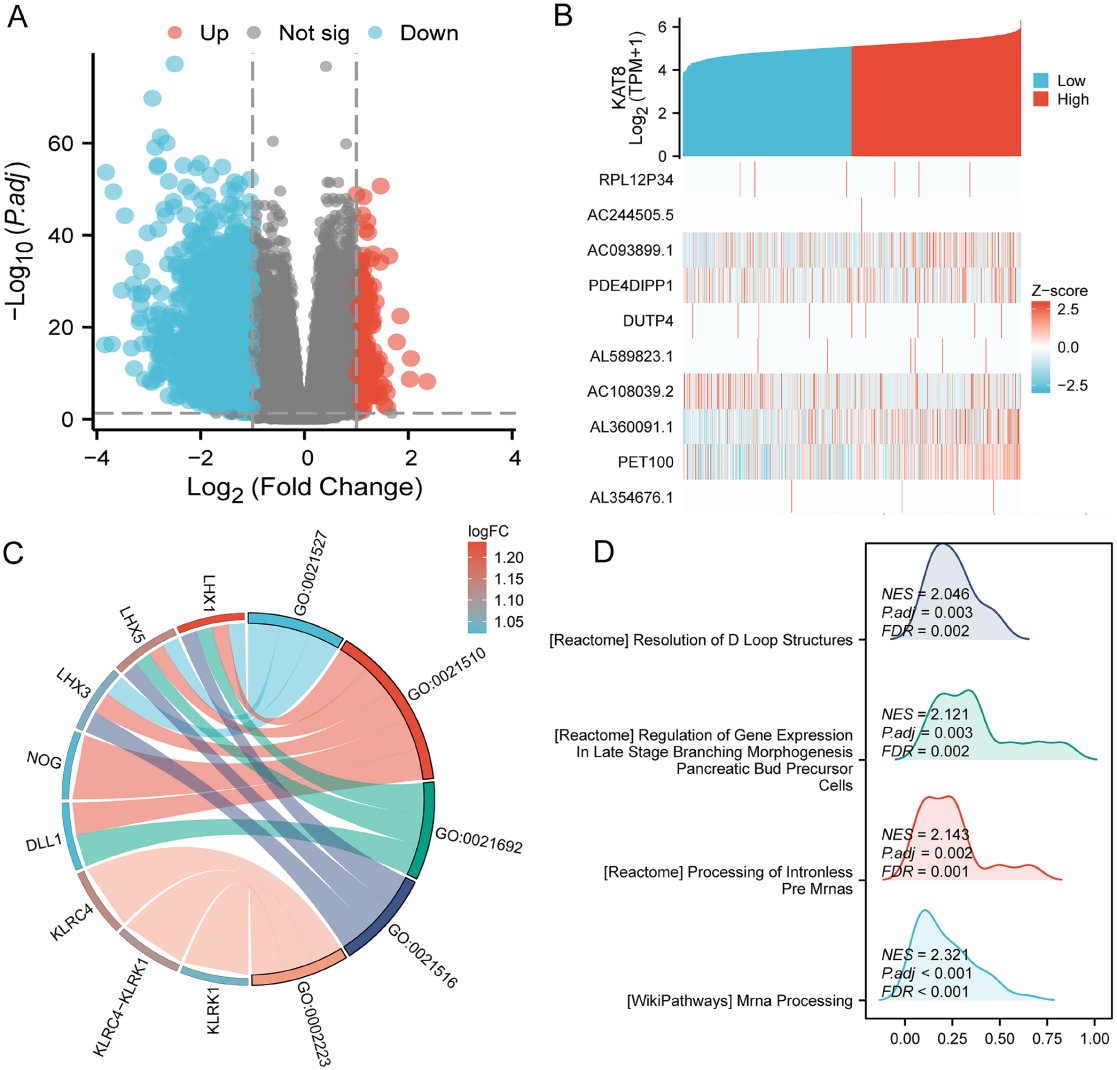
Functional annotation analysis of DEGs related to KAT8. (A) The vitally up‐ and down‐regulated DEGs in the Volcano plot related to KAT8 expression. (B) The top 10 DEGs positively correlated with KAT8 level. (C) KEGG, GO and GSEA (D) analysis of DEGs.

To investigate the function of KAT8 expression in glioma, a total of 701 glioma samples were categorised into two groups based on the KAT8 expression level. The low expression group (Low) consisted of the bottom 50% of samples (*n* = 350), while the high expression group (High) consisted of the top 50% of samples (*n* = 351). A total of 214 genes were found to be significantly differentially expressed between the High and Low KAT8 expression groups (|logFC| > 2 and *p*.adj < 0.05). The volcano plot (Figure 4) provides a visual representation of the statistical significance of these genes. The x‐axis represents the log fold‐change (logFC), while the y‐axis shows the adjusted *p*‐value (−log10(*p*.adj)). The differential expression profile was characterised by a number of up‐regulated and down‐regulated genes, which may offer insights into the molecular mechanisms underlying KAT8's role in gliomas.

Next, we focused on the top 10 genes with the most significant upregulation. The correlation between these genes and KAT8 expression was analysed using Spearman correlation analysis, and the results are presented in a heatmap. Additionally, a GO‐KEGG integrated functional clustering analysis was performed on these 10 genes. The analysis revealed that all these genes are enriched in BPs, particularly those related to the development and differentiation of neurons in the spinal cord. The top enriched GO terms included: Spinal cord association neuron differentiation (GO:0021527), which indicates a role in the differentiation of neurons specifically in the spinal cord. Spinal cord development (GO:0021510) reflects the involvement of these genes in the formation and maturation of spinal cord structures. Subsequently, GSEA demonstrated that mRNA and pre‐mRNA processing pathways were vitally enriched in the high KAT8 level group (Figure 4D).

### 3.6 Single‐Cell Sequencing to Analyse the Expression and Functional Relevance of KAT8 in Tumours

**FIGURE 5:**
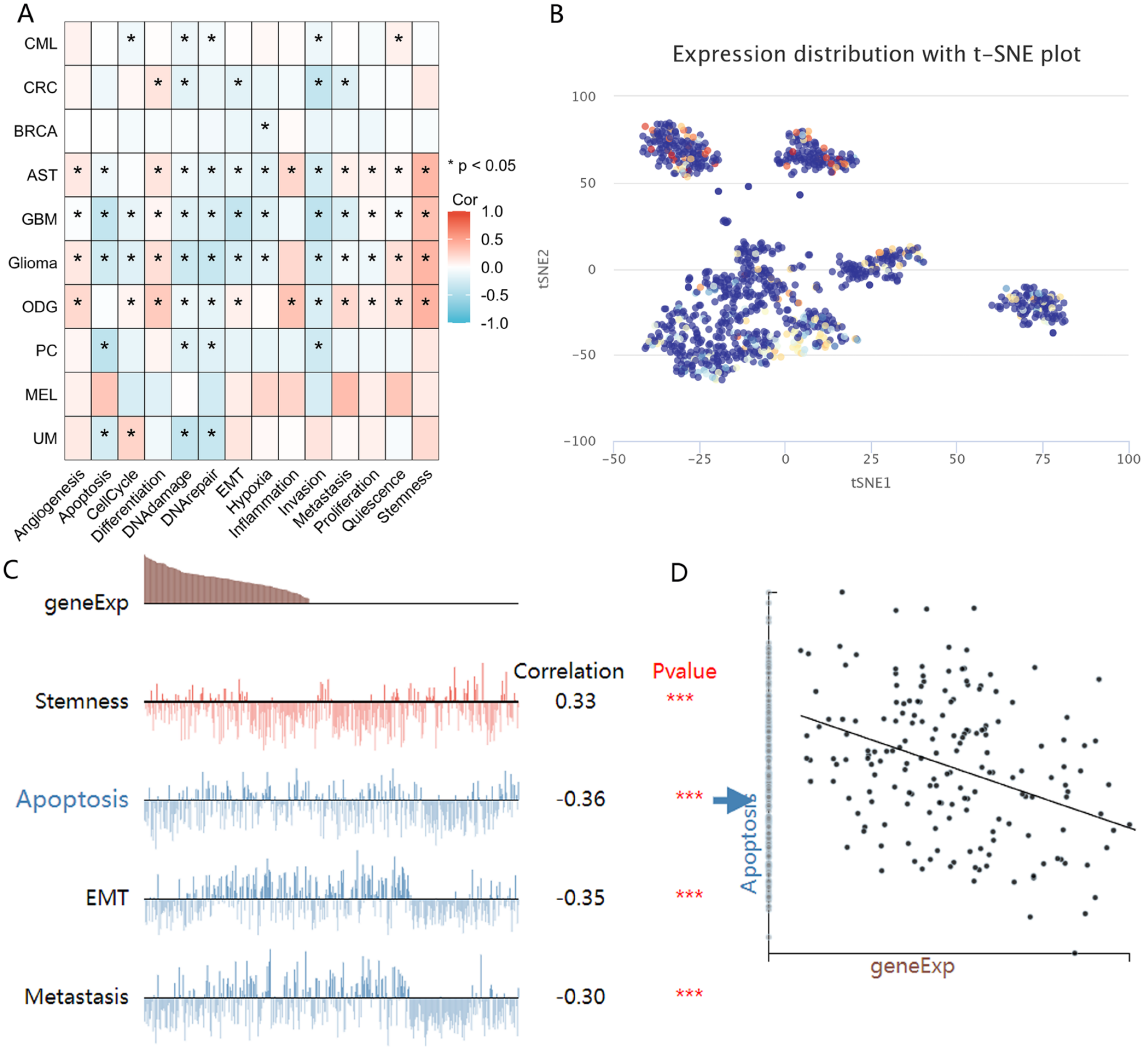
Expression pattern of KAT8 in single‐cell sequencing and its correlation with tumour functional status. (A) Heatmap showed the correlation between KAT8 expression and different tumour functional status based on CancerSEA database. (B) T‐SNE diagram demonstrated KAT8 expression profiles in Glioma single cells. (C) The relationship between KAT8 expression and apoptosis, stemness, EMT, and metastasis in GBM. (D) The correlation scatter plot demonstrating a negative relationship between KAT8 expression and apoptosis.

Since the complexity of tumour cell populations, single‐cell transcriptomic sequencing has emerged as a vital method for examining the diversity among cancer cells, immune cells, endothelial cells, and stromal cells. In our study, we validated the expression of KAT8 at the single‐cell level across various cancer types and explored its association with the functional status of tumours. The findings reveal that KAT8 expression is intricately linked to a diverse array of central nervous system tumours (Figure 5A). KAT8 expression within GBM exhibits a pronounced negative correlation with apoptosis. In astrocytoma (AST), KAT8 levels demonstrate a positive correlation with invasion characteristics. Furthermore, across all glioma subtypes, KAT8 is positively linked to stemness attributes. T‐SNE dimensionality reduction analysis unveiled significant heterogeneity in KAT8 expression levels within single‐cell sequencing of GBM (Figure 5B). Figure 5C showed the relationship between KAT8 expression and apoptosis, stemness, EMT, and metastasis in GBM. Figure 5D further corroborates KAT8 expression using a correlation scatter plot, demonstrating a negative relationship with apoptosis.

### 3.7 Relationship Between KAT8 Expression and Immune Infiltration in Glioma

**FIGURE 6:**
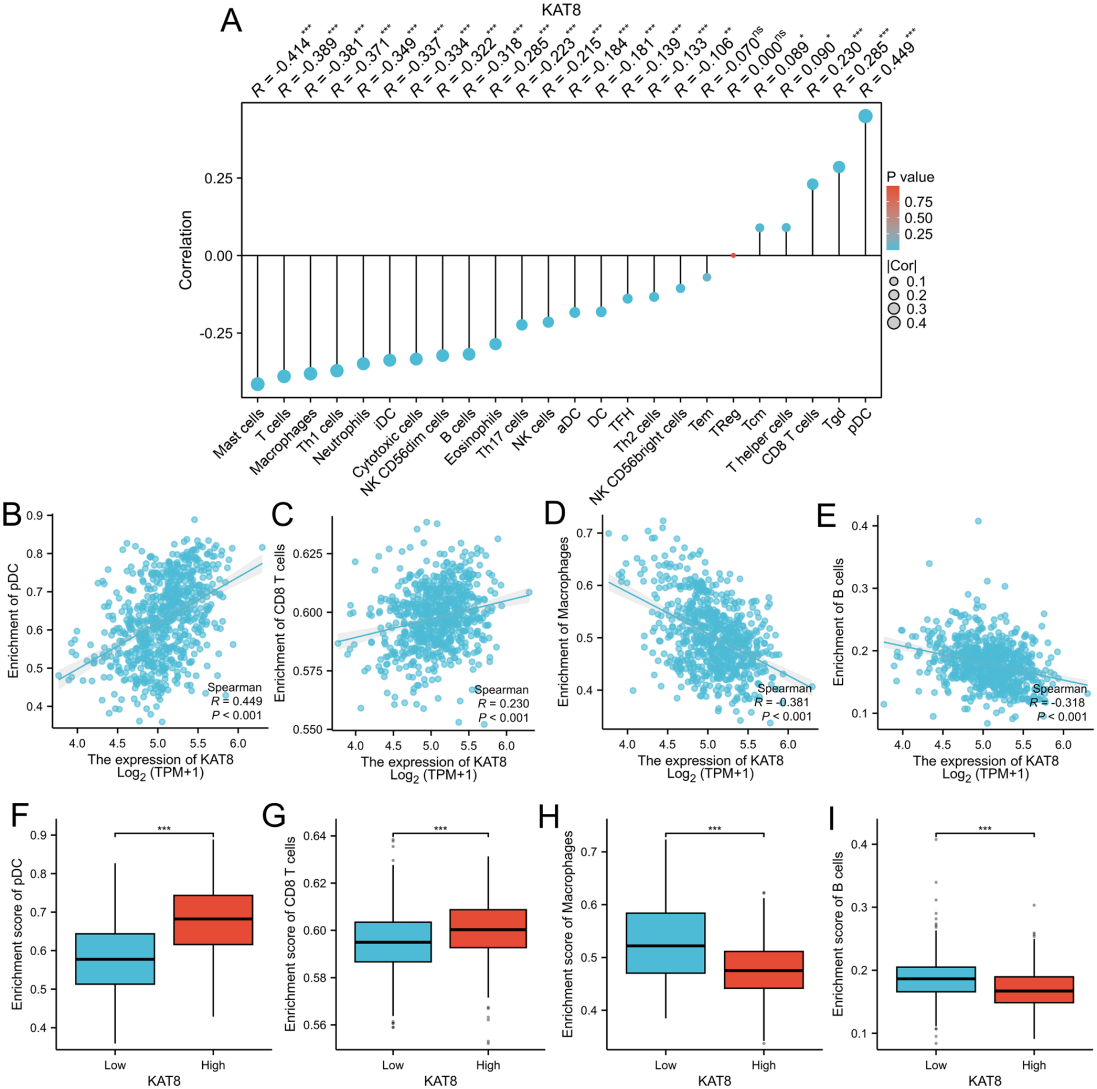
Relationship between KAT8 expression and immune infiltration in glioma. (A) A lollipop graph illustrating the association between KAT8 expression and immune infiltration levels. (B) Dot graphs comparing immune infiltration scores between high and low KAT8 expression groups. (C) Scatter graphs demonstrating the relationship between KAT8 expression levels and various immune cell populations. ***p < 0.001.

To explore the potential impact of lactylation on immune cell activity, we analysed whether KAT8 expression levels correlated with immune cell abundance in the tumour microenvironment (TME) using the TIMER database. As shown in Figure 6A–E, the lollipop plot indicated the predominantly positive correlation between KAT8 expression and five kinds of immune cells, including pDC (*R* = 0.449), Tgd (*R* = 0.285) and CD8 T cells (*R* = 0.23) (*p* < 0.001). KAT8 was negatively and moderately correlated with the infiltration level of macrophages (*R* = −0.381) and B cells (*R* = −0.318) (Figure 6D,E, *p* < 0.001). A further analysis showed that KAT8 expression influenced the infiltration of pDC and CD8 T cells, with significantly higher proportions in the high KAT8 expression group (Figure 6F,G, *p* < 0.001). Figure 6H,I showed that the enrichment score of macrophages and B cells was higher in the KAT8‐low expression group (*p* < 0.001).

### 3.8 Down‐Regulation of KAT8 Expression Mediated by EV‐A71 Infection Promotes Apoptosis in Glioma Cell Lines

**FIGURE 7:**
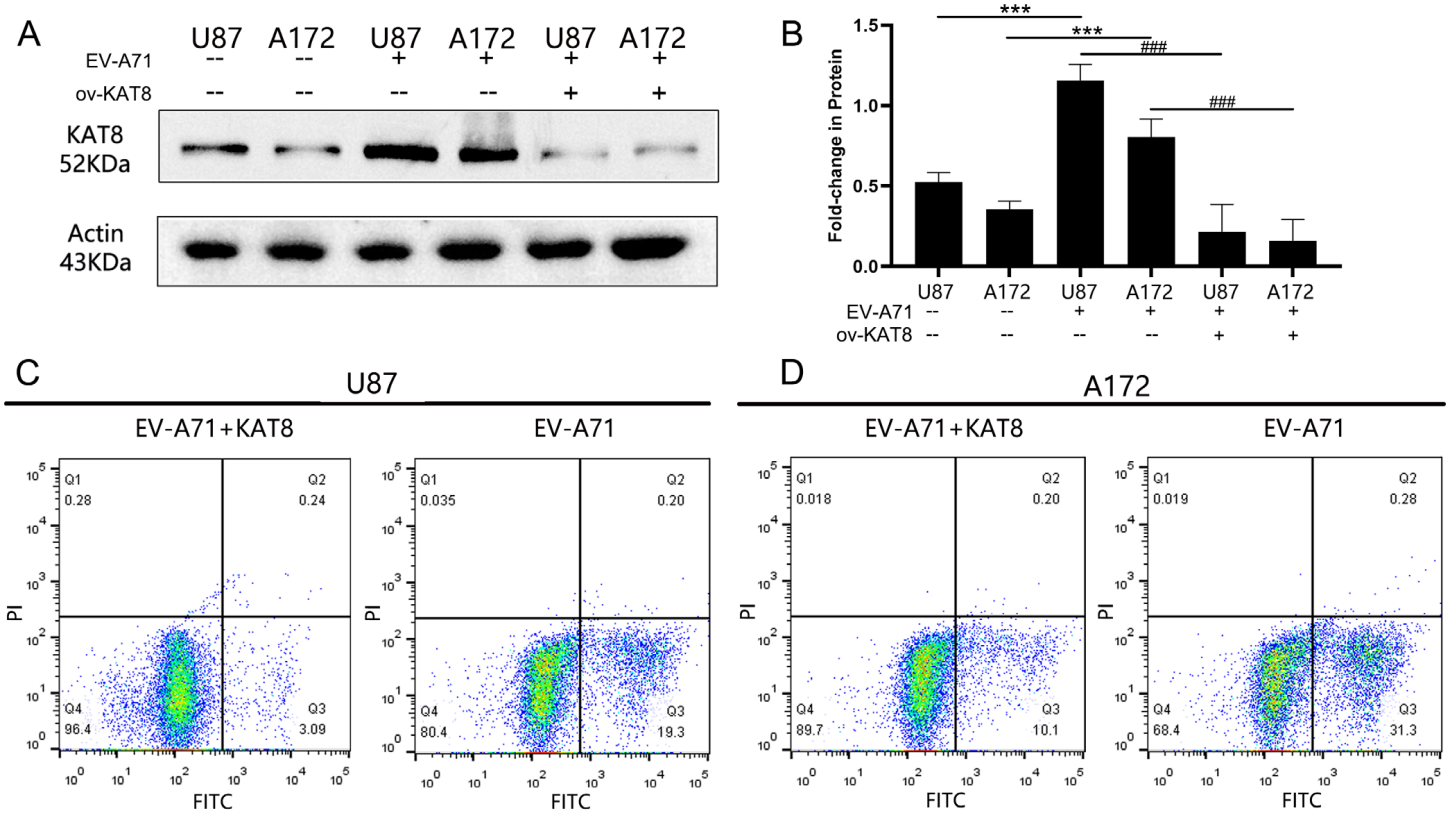
Down‐regulation of KAT8 expression mediated by EV‐A71 infection promotes apoptosis in glioma cell lines. (A, B) KAT8 protein expression level in U87 (lane1) and A172 (lane2) glioma cells. EV‐A71 infection resulted in a significant down‐regulation of KAT8 protein expression in U87 (lane5) and A172 (lane6) glioma cell lines. KAT8 plasmid transfection rescued the KAT8 expression in U87 (lane3) and A172 (lane4) glioma cell inducted by EV‐A71 infection. Down‐regulation of KAT8 expression mediated by EV‐A71 infection caused apoptosis in (C) U87 and (D) A172 glioma cells. While overexpression of KAT8 reversed this apoptosis. ***p < 0.001, ^###^ *p* < 0.001.

Based on the close relationship between KAT8 expression and glioma function, we next examined the expression of KAT8 in glioma cell lines. The results showed that KAT8 was highly expressed in U87 and A172 glioma cell lines (Figure 7A,B, lane1 and 2). To verify the function of KAT8 mediated by EV‐A71 infection in glioma cells, we infected U87 and A172 cells with EV‐A71. The results showed that the infection resulted in a significant down‐regulation of KAT8 protein expression in both cells at 48 h post‐infection (Figure 7A,B, lane5 and 6). While the expression of pLeGFP‐N3‐KAT8 rescues the KAT8 protein expression (Figure 7A,B, lane3 and 4). The single‐cell sequencing results in the previous section suggested that KAT8 functions most closely with apoptosis in gliomas. Therefore, we examined the apoptosis of two cells after the EV‐A71 infection or infection with KAT8 expression. The results showed that down‐regulation of KAT8 expression mediated by EV‐A71 infection caused apoptosis in both U87 (Figure 7C) and A172 (Figure 7D) cells. The apoptotic was mainly dominated by phosphatidylserine ectopia, which was obvious early apoptosis. The rate of early apoptosis was 19% in U87 cells and 31% in A172 cells. Neither cell showed significant late apoptosis. While overexpression of KAT8 reversed this apoptosis (Figure 7C,D).

## 4 Discussion

The association between KAT8 expression and various clinicopathological features provides insights into its biological significance in gliomas. Here we screened KAT8 as one of the most dramatically down‐regulated genes during oncolytic viruses EV‐A71 treatment. The expression of KAT8 is significantly higher in glioma patients with 1p19q co‐deletion compared to those without 1p19q co‐deletion. The higher KAT8 expression in the Codel group could reflect a metabolic shift or altered cellular behaviour in glioma, which may contribute to their distinct clinical outcomes. These findings imply that KAT8 may be involved in metabolic processes that are altered in tumours with 1p19q co‐deletion, potentially providing a biomarker for prognosis and therapy selection in glioma.

Patients younger than 60 years exhibited higher expression levels of KAT8 compared to their older counterparts. This result may suggest a younger age‐related metabolic or cellular phenotype that drives KAT8 expression, possibly linked to a more aggressive or distinct tumour biology in younger patients. This trend aligns with studies that have shown that younger patients often have a higher metabolic turnover in tumour cells, which might lead to increased lactate production and thus higher KAT8 expression [19].

The down‐regulation of KAT8 in grade IV gliomas might be reflective of altered metabolic states associated with tumour aggressiveness and poor prognosis . This finding aligns with existing literature that suggests metabolic changes in high‐grade gliomas, such as reduced reliance on oxidative phosphorylation and increased glycolytic activity, may suppress certain enzymes like KAT8 [20]. However, the absence of a significant difference between grades I–III could indicate that KAT8 expression is not a reliable discriminator for tumour grade in lower‐grade gliomas, pointing to the need for further investigation into its specific role in glioma progression.

Patients with high or low KAT8 expression exhibited similar survival outcomes, suggesting that KAT8 expression may not have a direct impact on prognosis in lower‐grade gliomas. However, high KAT8 expression is associated with a significantly higher survival rate in grade IV gliomas. This finding suggests that KAT8 may play a distinct role in high‐grade gliomas, potentially mitigating tumour progression or altering the tumour microenvironment to benefit patient survival. Therefore, high KAT8 expression in grade IV gliomas may provide valuable prognostic information and guide the development of therapeutic strategies. This protective effect, confirmed by multivariate Cox analysis, suggests that KAT8 might play a complex, context‐dependent role in glioma biology. It may be involved in pathways that counteract the aggressive nature of high‐grade gliomas, possibly through regulation of cell differentiation or apoptosis.

In cancer treatment, resistance can arise through genetic mutations in cancer cells, allowing them to evade the effects of chemotherapy or targeted therapies [21]. To date, as a promising therapy, oncolytic virotherapy significantly improves the survival of patients with tumours [22]. An oncolytic virus is engineered to selectively infect and destroy cancer cells while sparing normal cells, potentially reducing the harm caused by traditional treatments [23]. These viruses target cancer cells through specific surface proteins that bind to receptors more abundant on cancer cells, ensuring specificity. In 1991, Martuza et al. first found that intraneoplastic inoculation of herpes simplex virus‐1 with thymidine kinase‐negative mutant inhibited glioma tumour growth in nude mice and prolonged survival time [24]. Zhang et al. in 2020 illustrated the oncolysis activity of enterovirus A71 (EV‐A71) in glioma and elucidated that miR124‐sensitive EV‐A71 decreased the potential neurotoxicity [25]. Our present study revealed that EV‐A71 infection could be a powerful enhancer of cell apoptosis by increasing KAT8 expression levels in glioma.

The functional annotations and single‐cell sequencing analyses provide insights into the potential mechanisms of KAT8 action in gliomas. In the GO‐KEGG integrated functional clustering analysis, a significant association was observed between the identified genes and BP, particularly those related to the development and differentiation of neurons in the spinal cord [26]. These findings suggest that the identified genes are crucial in neurodevelopmental processes, particularly those governing the differentiation, growth, and structural organisation of neural tissues. The prominent enrichment in spinal cord and cerebellar development suggests a potential link to neurological diseases or developmental disorders associated with these regions, such as spinal cord injury or cerebellar ataxia [27]. The negative correlation between KAT8 expression and apoptosis in GBM cells, coupled with the increased apoptosis observed in oncolytic virus infection, indicates a potential anti‐apoptotic function. This contradicts its association with better prognosis and highlights the complex nature of KAT8's role in glioma biology. The negative correlation between KAT8 expression and apoptosis in glioblastoma cells, as revealed by single‐cell sequencing, was further validated by our in vitro experiments showing increased apoptosis upon KAT8 knockdown in glioma cell lines. Our study offers important insights into the role of KAT8 in glioma cell apoptosis and EV‐A71 infection. However, there are several limitations that need to be acknowledged. One limitation is the lack of validation through in vivo animal studies. Additionally, there is an absence of data regarding the regulatory mechanisms of EV‐A71 infection within glioma cells. In future research, we plan to investigate how EV‐A71 infection influences glioma regulation pathways.

In summary, our findings highlight KAT8 as a significantly down‐regulated gene during oncolytic virus infection. It has a promising target for further research in glioma biology and potential therapeutic interventions. The differential expression and prognostic significance of KAT8 across glioma grades suggest that targeting KAT8 or its associated pathways could offer new avenues for glioma treatment, particularly in high‐grade tumours where its expression correlates with improved survival.

## Author Contributions

**Xiaofeng Yin:** conceptualization (equal), data curation (equal), formal analysis (equal), methodology (equal), resources (equal), validation (equal), visualization (equal), writing – original draft (lead), writing – review and editing (equal). **Zhen Hao:** formal analysis (equal), methodology (equal), resources (equal), software (equal), writing – original draft (lead), writing – review and editing (equal). **Qi Liu:** formal analysis (equal), methodology (equal), resources (equal), visualization (equal), writing – review and editing (equal). **Rui Ding:** formal analysis (equal), investigation (equal), resources (equal), visualization (equal). **Laizhao Chen:** data curation (equal), formal analysis (equal), resources (equal), software (equal), visualization (equal). **Mingliang Jin:** conceptualization (equal), formal analysis (equal), validation (equal), visualization (equal), writing – review and editing (equal). **Songquan Wang:** conceptualization (equal), formal analysis (equal), investigation (equal), resources (equal), software (equal), validation (equal), visualization (equal), writing – original draft (equal), writing – review and editing (equal).

## Conflicts of Interest

The authors declare no conflicts of interest.

## Supporting information


**Table S1.** Down‐regulated genes after EV‐A71 infection.

**Table S2.** Up‐regulated genes after EV‐A71 infection.

## Data Availability

The data that support the findings of this study are openly available at https://www.ncbi.nlm.nih.gov/, reference number PRJNA562271.
